# Assessment of multiple pharmacological mechanisms in the ascaris sensitive sheep model of allergic asthma

**DOI:** 10.1186/1476-9255-10-S1-P15

**Published:** 2013-08-14

**Authors:** Michael Caniga, Janice D Woodhouse, Alan Wilhelm, Malgorzata A Gil, Robbie McLeod, Lily Y Moy, Michael A Crackower, Thomas Miller, William M Abraham, Milenko Cicmil

**Affiliations:** 1 Merck Research Laboratories, Boston, MA 02115, USA; 2 Mount Sinai Medical Center, Miami Beach, FL 33140, USA

## Background

Asthma is a multifaceted disease that presents with a combination of reversible bronchoconstriction, inflammation, and airway remodeling. Historically, a variety of in vivo models have been used by preclinical investigators as surrogates for the disease. However, many of these models have significant limitations. For example, rodent models relying solely on TH2 mediated respiratory inflammation have inadequate predictability and clinical translatability because they can only recapitulate partial aspects of the human disease. In order to align with human Phase I allergen challenge experiments we used ascaris sensitive sheep model. This model enables us to investigate role of different mechanisms on early and late asthmatics responses.

## Methods and materials

After inhaled ascaris antigen challenge, sheep display a classical airway response featuring an early airway response (EAR), a late airway response (LAR), and airway hyperreactivity (AHR), as described by Abraham et al. (1983) [1]. Compound can be administered either topically (via inhalation) or systemically prior to the inhaled challenge. A range of compounds and mechanisms have been investigated in this model.

## Results

Standard of care anti-inflammatory compounds such as corticosteroids and leukotriene inhibitors show little inhibition of the EAR at low doses but completely inhibit the LAR and AHR. Novel anti-inflammatory targets such as Chemoattractant receptor-homologous molecule expressed on T-helper type 2 cells (CRTH2) inhibitor, MK-7246, also show little inhibition of the EAR at low doses and blocked the LAR and AHR. Broncho-constrictors like the Β2-adenergic antagonist, fomoterol, greatly inhibit the EAR (~85%) but have less of an effect on the LAR (~60%).

## Conclusion

Cumulative data from this model has shown to be a good predictor for Phase 1 allergen challenge trial. Standard of care compounds and novel mechanisms used perform similarly in human trials as they did in ascaris sensitive sheep model. This model offers some key element of airways reactivity function similar to human asthma.
